# Implications of photodynamic cancer therapy: an overview of PDT mechanisms basically and practically

**DOI:** 10.1186/s43046-021-00093-1

**Published:** 2021-11-15

**Authors:** Nafiseh Sobhani, Ali Akbar Samadani

**Affiliations:** 1 Department of Basic Medical Sciences, Neyshabur University of Medical Sciences, Neyshabur, Iran; 2 Healthy Ageing Research Center, Neyshabur University of Medical Sciences, Neyshabur, Iran; 3 Clinical Research Development Unit of Poursina Hospital, Guilan University of Medical Sciences, Rasht, Iran

**Keywords:** Photodynamic cancer therapy, Mechanisms, Oncological studies, Cancerous tissues and cells

## Abstract

**Background:**

Tumor eradication is one of the most important challengeable categories in oncological studies. In this account, besides the molecular genetics methods including cell therapy, gene therapy, immunotherapy, and general cancer therapy procedures like surgery, radiotherapy, and chemotherapy, photodynamic adjuvant therapy is of great importance. Photodynamic therapy (PDT) as a relatively noninvasive therapeutic method utilizes the irradiation of an appropriate wavelength which is absorbed by a photosensitizing agent in the presence of oxygen.

**Main body of the abstract:**

In this procedure, a series of events lead to the direct death of malignant cells such as damage to the microvasculature and also the induction of a local inflammatory function. PDT has participated with other treatment modalities especially in the early stage of malignant tumors and has resulted in decreasing morbidity besides improving survival rate and quality of life. High spatial resolution of PDT has attracted considerable attention in the field of image-guided photodynamic therapy combined with chemotherapy of multidrug resistance cancers. Although PDT outcomes vary across the different tumor types, minimal natural tissue toxicity, minor systemic effects, significant reduction in long-term disease, lack of innate or acquired resistance mechanisms, and excellent cosmetic effects, as well as limb function, make it a valuable treatment option for combination therapies.

**Short conclusion:**

In this review article, we tried to discuss the potential of PDT in the treatment of some dermatologic and solid tumors, particularly all its important mechanisms.

## Highlights

1. The implication of PDT is approved clinically and is a noninvasive therapeutic method that can employ an elective cytotoxic function toward cancerous tissues.
2. The effectiveness of PDT in cutaneous malignancies as a promising treatment modality and in some cases like lung cancer has been proven.
3. Designing methods to overcome constraints such as photosensitivity, poor light penetration, low tumor selectivity, and systemic toxicity seems to be effective in PDT efficiency.
4. PDT leads to a sequence of photochemical and photobiologic activities that leads to irreversible photo damage to cancerous cells.

## Background

### Main text

Photochemical treatment of cancer, often called photodynamic therapy (PDT), is a relatively noninvasive method compared with other common cancer therapy modalities in treatment of cancerous small tumors. Using PDT as an adjuvant treatment was recommended in literatures to overcome some obstacles of common monotherapies such as surgery, chemotherapy, and radiation therapy [106, 131, 134]. In some researches, high spatial and temporal resolution of PDT has been reported as a functional feature in image-guided PDT [59, 124, 127, 132]. This method is based on the reactions of a photosensitizer in the presence of oxygen molecules and appropriate wavelength of light [8, 74, 105].

The performance of PDT requires some qualifications including (1) a photosensitizer (PS) with maximum tumor uptake, (2) sufficient elapsed time after injection to achieve most accumulation of the photosensitizer in the tumor, and also (3) irradiation of light with appropriate wavelength to optical destruction of the tumor.

There are two oxidative mechanisms in optical destruction of tumor cells. Photosensitizer interacts with a biomolecule or oxygen, and free radical production occurs as a result of the transfer of electron or hydrogen. Singlet oxygen produces by energy transfer from triplet excitation mode to triplet ground state of oxygen molecule.

Different types of biomolecules such as unsaturated fats, cholesterol, and alpha amino acids such as tryptophan 3 and metanil 6 react with singlet oxygen easily. These compounds are the main components of different biological membranes. So, the membrane damage is an important process which causes the necrosis and destruction of blood vessels through PDT [14]. The photosensitizer usually is injected with aqueous buffer solution or liposome, and light source is often a laser with optical fibers to optimal light transfer to the therapeutic region.

### Physics of photodynamic therapy

Several phenomena such as scattering, reflection, transmission, and absorption may occur after tissue irradiation, which determine the type of effect and depth of penetration in tissue [91].

To perform a biological reaction, photons must first be adsorbed by a photosensitizer, and this is possible when the wavelength of light is the same as the absorption spectrum of the photosensitizer [78]. Intense absorption in the wavelength less than 600 nm by in vivo pigments (mainly hemoglobin) and less efficiency of singlet oxygen production in the wavelength more than 900 nm sometimes eliminate the clinical applications to the region of 600–900 nm [78].

### Mechanism of competitive reactions in photodynamic therapy

**Fig. 1:**
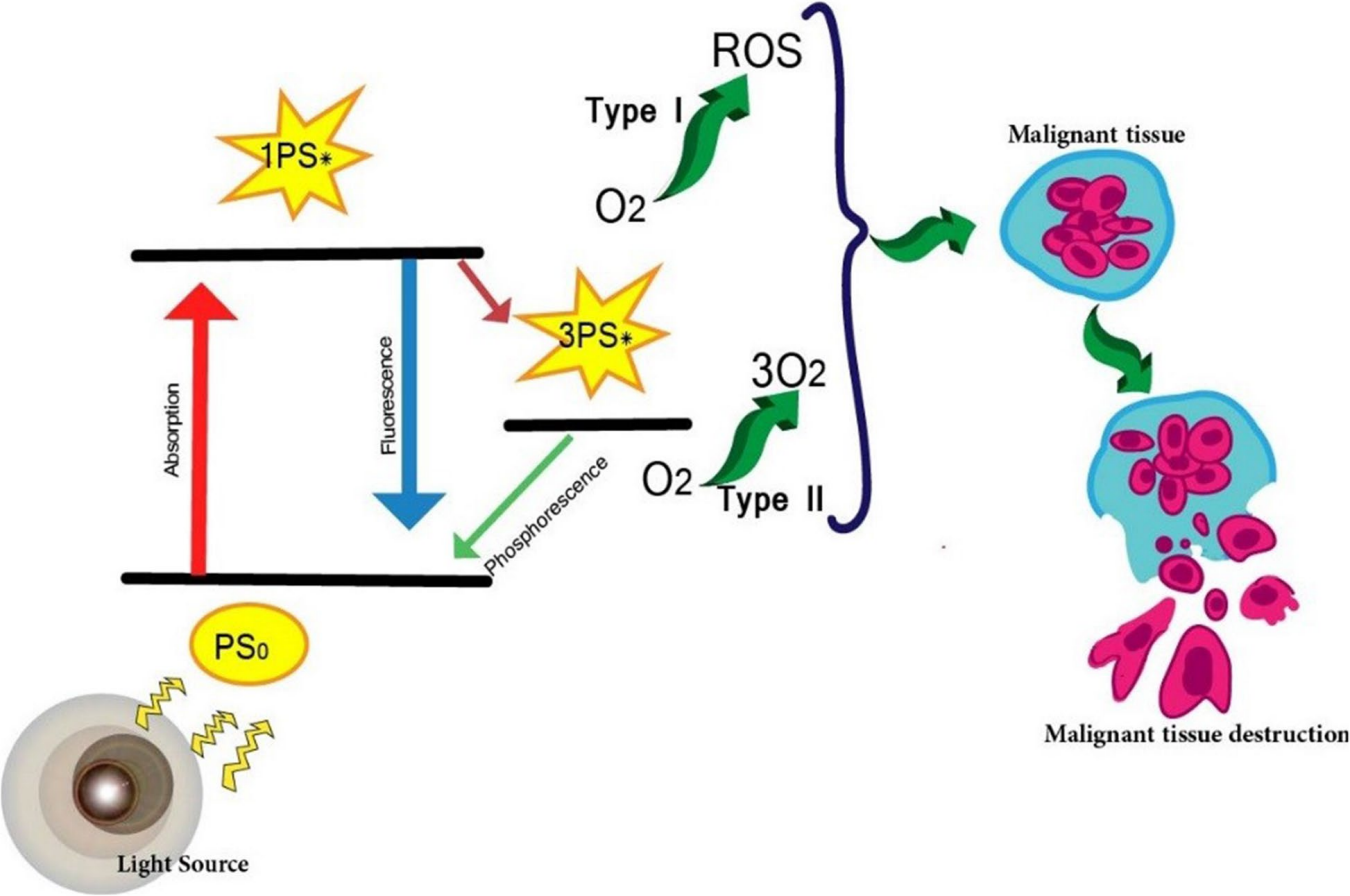
Reaction types I and type II of photosensitizer application in destroying the malignant tissues with the involvement of oxygen species (ROS) in photodynamic therapy

Following the absorption of a photon with appropriate energy, the photosensitizer molecule is transferred from the ground state (S_0_) to excited state (S_1_) which may return to its primitive ground state along with fluorescence radiation or be transferred to triplet excited state as an inter-system crossing. The triplet state of photosensitizer molecule in the tissue has a relatively long lifespan which can cause changes in surrounding molecules and initiate two competitive reactions called reaction types I and II (Fig. 1) [10, 24].

Reaction type I includes the transfer of electron or proton to oxygen and surrounding molecules to form anionic or cationic radicals. These radicals can react with oxygen molecule to form the reactive oxygen species (ROS). Reaction type I often leads to the formation of superoxide ions by the transfer of one electron to an oxygen molecule. These ions do not act as active ions in biological systems, but they can produce hydrogen peroxide (H_2_O_2_), which is easily absorbed from cell membranes. In high concentrations, H_2_O_2_ can react with super oxide molecules caused by the production of hydroxyl as an active radical which has the ability to ionize any molecules with low activation energy.

In reaction type II, the photosensitizer molecule, by transitioning from the triplet state to ground state and transfer of energy to oxygen molecule, converts it to excited singlet oxygen. The singlet oxygen as a chargeless molecule can spread to the cytoplasm and biological membranes. Approximately all of photosensitizers have high quantum yield in this reaction.

Photosensitizers typically produce one singlet oxygen per every two absorbed photons. The available evidences show that the singlet oxygen is the main intermediary of biological damage in PDT. Some studies have shown that the tissue necrosis needs to be 10^18^–10^19^ singlet oxygen per cm^3^ [122].

Reactions types I and II as direct effects of PDT occur in parallel depending on the type of photosensitizer and the oxygen concentration. It should be noted that for more applied photosensitizers, reaction type II is a dominant process [91].

PDT assists in tumor destruction via apoptosis and necrosis in direct tumor cell killing, hypoxia and starvation of tumor in vasculature damage and T cells, antibodies, and long-term memory immunity in stimulation of the immune system [69, 97].

### Photosensitizers

There is no history of photodynamic therapy that can be said without regard to hematoporphyrin. For the first time, Schere produced the impure hematoporphyrin, and its fluorescence spectrum was interpreted by Thudichum in 1867 [11, 50]. After the identification of impurity of hematoporphyrin, researches began to find products with higher purity such as tetraphenylporphine sulfonate (TPPS), phthalocyanine (Pc), aluminum phthalocyanine sulfonates (AlPcS), meta hydroxyl-phenyl (mTHPC), and protoporphyrin IX.

The first attempts to apply PDT in tumor treatment and other skin diseases such as lupus were made by Tappeiner’s group in 1903–1905. They intratumorally injected some of useful dyes such as eosin, fluorescein, and sodium dichloroanthracene disulfonate, and satisfactory results were reported. Then, much research have been done on the sensitivity of materials to light which showed that the presence of oxygen is necessary for the occurrence of a photodynamic effect [63].

Primary sensitizers had three main drawbacks: skin sensitization, low selectivity, and poor absorption in infrared region. So, deep tumor treatment was difficult [14]. A suitable photosensitizer should have the following characteristics:

A. Photophysical property: high absorption in the wavelengths of 630–980 nm to maximum penetration in tissue and minimum absorption in the range of 400–600 nm which causes photosensitivity by sunlight (Table 1).
B. Photochemical property: high singlet oxygen production to maximum PDT efficiency. Photosensitizer also must be fluorescent for biological distribution monitoring via spectroscopy.
C. Chemical property: high stability, cheap and convenient synthesis, solubility in water, and participation in the body kinetic cycle without the need to release intermediary such as liposomes and emulsions.
D. Biological property: low toxicity, rapid clearance from vascular system, selective absorption in tissue, and penetration into micron-size cellular targets [1, 38, 45, 50, 52, 77, 80, 83, 122].

**Table 1:** Penetration depth of different wavelengths of light applied in PDT

Wavelength (nm)	Penetration depth (cm)	Reference
NIR region	< 10	[25]
662–780	0.4–1	[7]
597–622	0.3–0.4	[7]
577–597	0.3	[7]
492–577	0.2	[7]
455–492	0.1	[7]
390–455	0.1	[7]

Some photosensitizers such as 5-aminolevulinic acid (Levulan), methyl aminolevulinate (Metvix), hexyl 5-aminolevulinate (Hexvix), and porfimer sodium (Photofrin) were approved by the FDA for clinical application of PDT [36].

A large number of new photosensitizers are synthesized via changes in structure of various compounds such as chlorines, bacteriochlorines, phthalocyanines, texarphions, and porphyrins.

### Deep tumors treatment by PDT

Despite the advantages of photodynamic therapy, this method faces obstacles in the treatment of deep tumors using short wavelengths of light. Light with a range of visible and ultraviolet energy due to autofluorescence and scattering by biological systems cannot penetrate into deep tissues and limits treatment to tumors within a few millimeters of the tissue surface [5, 23, 56, 60, 116, 119].

The penetration depth increases as the wavelength of the incoming light increases and refers to the area that receives 37% of the incoming light. For example, the penetration depth of light with a wavelength of 693 nm in bladder tissue is approximately 40% greater than the penetration depth of light with a wavelength of 633 nm [101]. Meanwhile, the use of near-infrared (NIR) wavelengths in the biological windows I (650–950 nm) and II (1000–1350 nm) makes it possible to treat tumors located at greater depths due to the reduction in autofluorescence and tissue dispersion in this area. So, there is a need for photosensitizers with absorption in this range of energy.

While the absorption wavelengths of most of photosensitizers are in the visible and ultraviolet regions, the amazing properties of some sensitizers and nanoparticles promise to use NIR light in the treatment of deep tumors.

### NIR photosensitizers

Many advances in therapies have further limited barriers of NIR sensitizers such as low water solubility, low quantum yield, low stability in biological environments, low light detection sensitivity, and optical stability [60]. For the first time in Canada, the use of Photofrin with an absorption wavelength of 630 nm in the treatment of bladder tumors was an important event in photodynamic therapy.

Cyanine, squaraine derivatives, and BODIPY (borondipyrromethane) are the most important NIR adsorbent sensitizers used in clinical imaging trials. Porphyrins such as 3- (1′-butyloxy) ethyl-3-deacetyl-bacter-iopurpurin-18-N-butylimide methyl ester with significant absorption in the tumor and low optical toxicity in the skin was proposed as a functional agent in imaging and photodynamic therapy [87, 130]. Bromo-substituted BODIPY with high singlet oxygen quantum yield is suitable for the photodynamic treatment of deep-seated tumors [126]. Because of singlet oxygen production by porphyrin and phthalocyanine derivatives, meso-tetraarylporphyrins, core modified porphyrin dendrimers, and bacteriochlorines are recommended candidates for NIR-triggered photodynamic therapy [60]. One of the obstacles of PDT performance of these photosensitizers is the fluorescence quenching due to their aggregation in biological environments. Although some PSs with aggregation-induced emission (AIE) are introduced to overcome this limitation, their poor molar extinction coefficient and photobleaching feature made researchers interested in the application of nanoparticles in NIR-PDT [20, 41, 117, 120].

Some nanostructures based on gold, palladium, carbon, copper selenide, tungsten oxide, etc., are suggested in literatures as functional theranostic particles in NIR-PDT and fluorescence imaging [56, 115]. Among them, metallic gold nanostructures have been noted for their non-toxic and desirable optic nature (tunable localized surface plasmon resonances (LSPR)). To this end among the several gold nanoparticle morphologies, Au nano-echinus structures with high extinction coefficient of ∼10^12^ M^− 1^ cm^− 1^ in the NIR region seem to exhibit excellent PDT efficiency in both biological windows I and II [104, 114].

### Biological targets in photodynamic therapy

The type of photosensitizer and other parameters such as irradiation rate, time interval, and total dose of treatment are tunable for the selection of biological targets. Unlike radiotherapy, the main purpose of PDT is not to damage DNA, but instead of the prevention of cell proliferation, somatic death occurs [81]. In photodynamic therapy of some tumors such as prostate, photosensitizers also can act as tumor oxygen eliminators via vasculature targeting.

Tissue responses in PDT are very rapid and visible, even before the treatment is completed.

Photosensitizers are localized in the mitochondria, cytosol, cytosolic membranes, Golgi apparatus, plasma membrane, endoplasmic reticulum, mitochondria, lysosome, and endosome, and more selective tumor uptake is related to the differences in the physiology of normal and neoplastic tissues [62]. Tumors have more interior volume, permeable vascular system, lipoprotein receptors, and less extracellular pH and lymphatic drainage than normal tissues [16, 129].

### Cell death pathways in photodynamic therapy

**Fig. 2:**
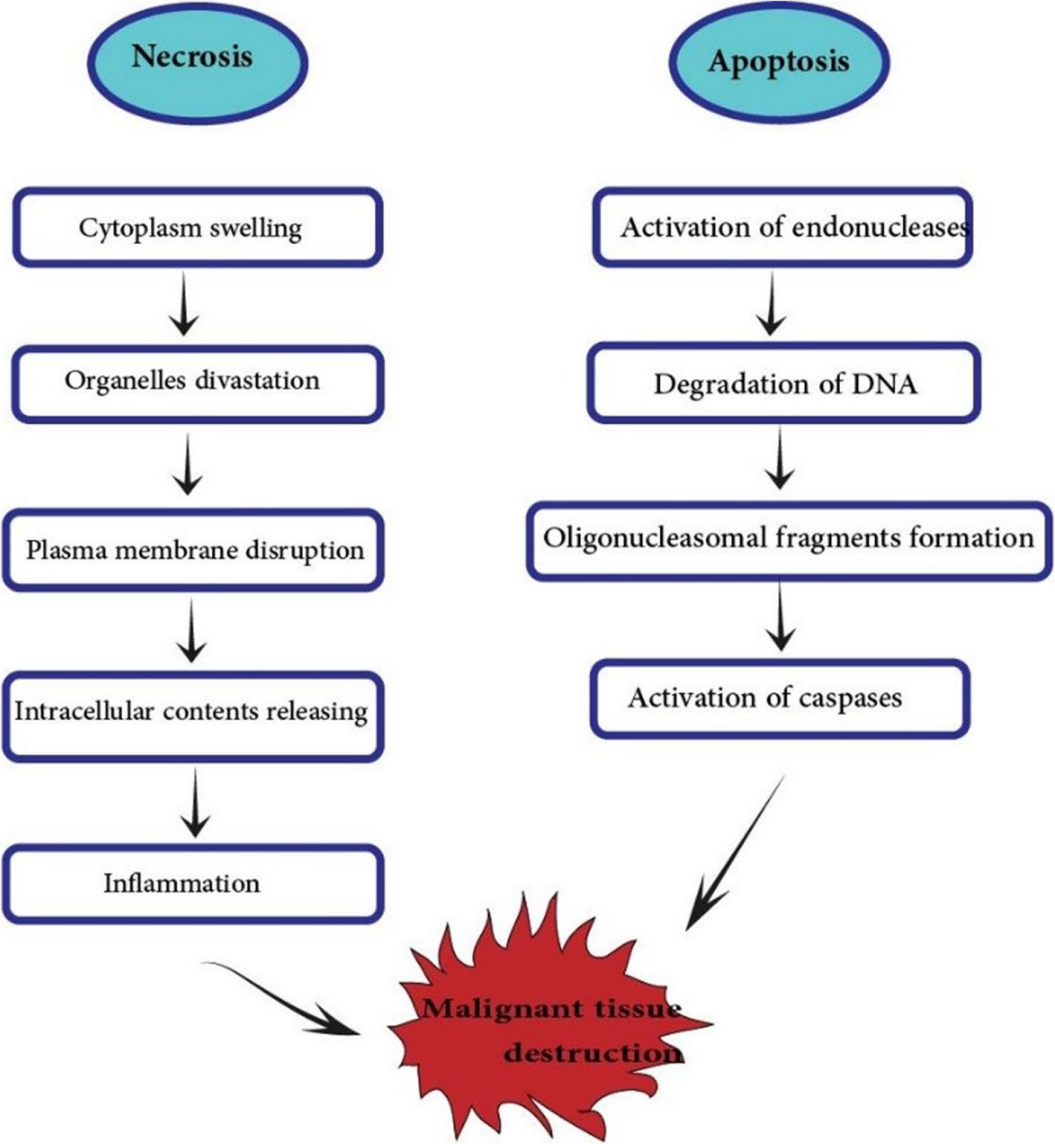
Mechanisms of necrosis and apoptosis induced by PDT

During PDT, oxidative stress in endoplasmic reticulum and photo oxidative cell damage cause the two modes of cell death, necrosis and apoptosis (Fig. 2).

After the release of extracellular proteins outside the cell, the activation of immune cells, migration to the site of cellular damage, and phagocytosis of damaged cells are the next events leading to antigen presentation and T cell activation [9].

Necrosis and apoptosis are determined by the properties of photosensitizer, cell line, irradiance wavelength, power density of radiation, and the oxygen concentration [19]. Necrosis is occurred following the cytoplasm swelling, release of intracellular contents, and inflammation. In apoptosis cell shrinkage, plasma membrane blebbing and nuclear fragmentation caused cell death [58].

Generally, photosensitizers which localized in the mitochondria induce apoptosis, and those in the lysosome elicit the apoptosis or necrosis response. In plasma membrane apoptosis, necrosis and rescue responses are involved. Rescue responses are accompanied with changes of genes and expression of protein [71].

According to the reports of literatures, in general, low-dose PDT leads to apoptosis while high dose PDT is followed by more necrosis [19].

### Cellular signaling mechanism

Proteins which are the most important factors in cell membrane signaling function are divided into transmembrane proteins (TM) and peripheral proteins. TM proteins with the ability to cross the membrane are included to receptors and transporters of the membrane. Ion channels are one of the membrane transporter categories with the role of neurotransmission and responsibility of cell signal transduction. Single-pass transmembrane receptors (SPTMRs) and G-protein-coupled receptors (GPCRs) also transmit the signals from the outside to the inside of the cells.

Membrane-binding domains (MBDs) such as homology-1(C1) and homology-2 (C2), protein kinase C (PKC), EEA1 (FYVE), and pleckstrin homology (PH) have the essential role in recruiting the peripheral proteins to the membrane during the membrane signaling process. Lipid anchored proteins, transmembrane receptors, and lipid-binding proteins are a few samples of proteins associated in signaling events [21].

After PDT, increasing the free calcium level within the cells occurs via Ca^2+^ entrance through ion channels, Ca^2+^ secretion in the endoplasmic reticulum (ER) and mitochondria, and ion exchange mechanisms. It can be followed by cell death or in certain conditions by survival [19].

**Fig. 3:**
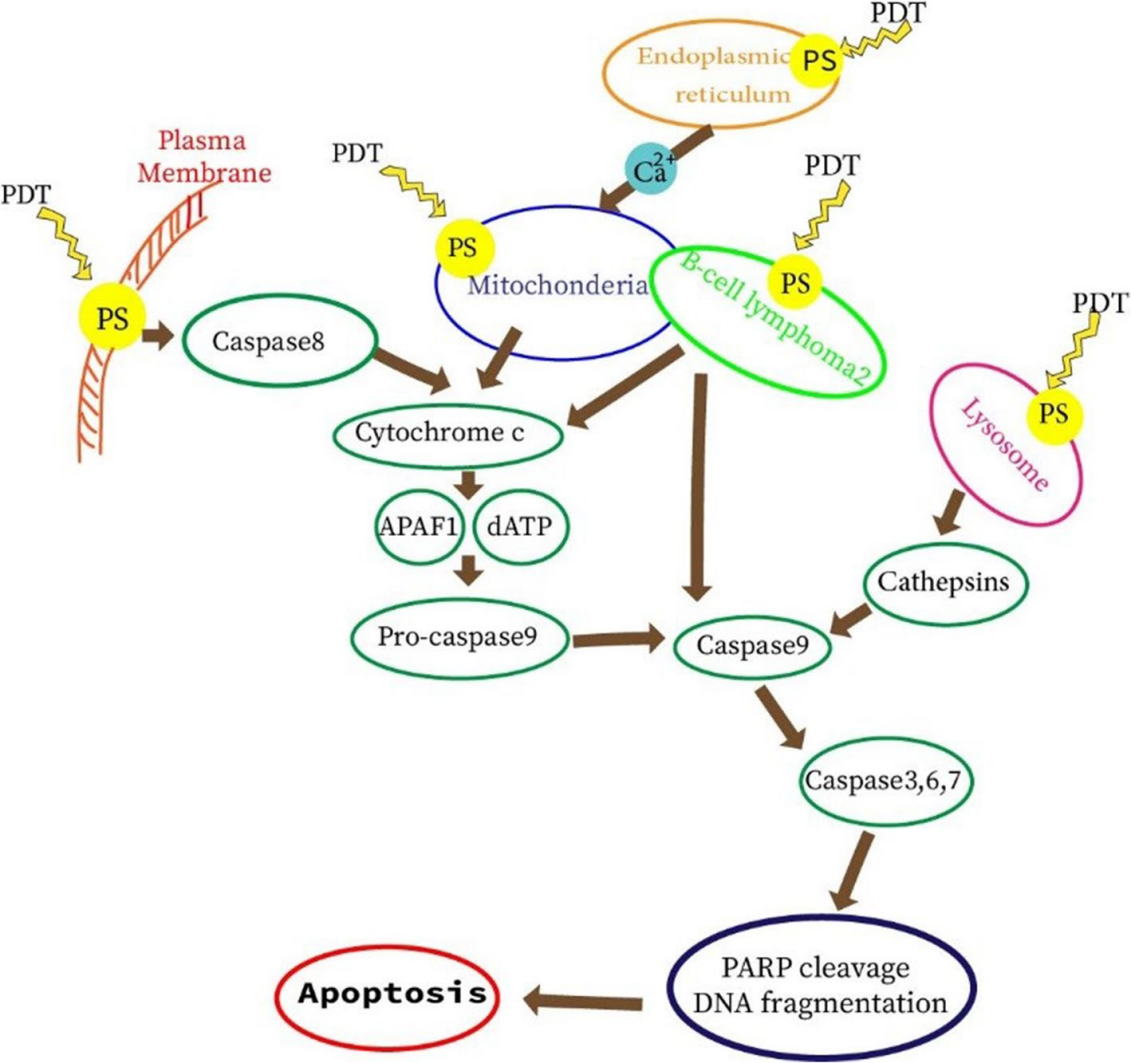
Cellular signaling pathways based on photosensitizer (PS) localization and apoptosis induction upon PDT

Apoptosis induced after PDT, by the mitochondrial localized photosensitizers such as benzoporphyrin derivative monoacid ring and silicone phthalocyanine Pc 4, is following some signaling pathways. After illumination, cytochrome c releases into the cytosol, and because of the onset of permeability of the mitochondria and releasing of the Ca^2+^, rapid drop in the mitochondrial membrane potential is observed upon PDT. Caspase 3, procaspase 3, apoptosis-activating factor-1 (APAF-1), caspase 9, and pro-caspase 9 are involved in the cleavage of DNA fragmentation factor (DFF) and poly (ADP-ribose) polymerase (PARP) enzyme (Fig. 3).

Some of signaling pathways belong to plasma membrane level. Phospholipase A2 (PLA2) and phospholipase C (PLC) enzymes are activated by PDT and participate in signal transduction. Releasing calcium from the internal stores also activate PLA2. In PDT of some cells such as T24 with hematoporphyrin derivatives, an increase in intracellular calcium leads to PLA2 activation and subsequently an increase in cAMP and prostaglandin E (PGE) with the important role in rescue response.

### Photodynamic therapy of solid tumors

The efficacy of PDT in treatment of different solid tumors was investigated in several studies. In terms of clinical effectiveness, method standardization, result reproducibility, and publication number, there is a specific and degreed treatment mythology in skin, esophageal, lung, and head and neck cancers. Cholangiocarcinoma, mesothelioma, brain tumors, and prostate and bladder cancers are in the second level, and gynecology, breast, pancreas, and intraperitoneal cancers are characterized in the third level.

Surgery is the preferred modality with no recurrence in treatment of patients with skin cancers except in multiple lesions or specific location tumors which are assigned to PDT [69]. Furthermore, in patients with immune system defects or Gorlin syndrome, PDT is more recommended than surgery. In fact, the first experiments using PDT were against skin tumors. It was because of the ease of use and the possibility of monitoring the results and treatment progress. 5-Aminolevulinic acid or ALA (Levulan), methyl aminolevulinate (Metvixia) (MAL), and aminolevulinic acid hydrochloride (Ameluz) are the common used photosensitizers for PDT of actinic keratosis. Vegter et al. have reported that ALA-PDT was the most effective method in photodynamic therapy of mild to moderate actinic keratosis of face and scalp [113]. Desired response to treatment with this method was observed in 75–89% of patients [69]. A recent randomized trial comparing four frequently used methods in the treatment of actinic keratosis reports patients who were treated with fluorouracil had lower disease recurrence than those who received imiquimod, MAL-PDT, or ingenol mebutate [39].

5-Aminolevulinic acid or ALA (Levulan) and methyl aminolevulinate (Metvixia) are used in squamous cell carcinoma (SCC) PDT and have more clearance rate than cryotherapy and 5-fluoracil (5-FU) [129].

Jansen et al. in a 5-year randomized control trial found more treatment efficacy of basal cell carcinoma (BCC) in 5% imiquimod cream compared to both MAL-PDT and 5-FU [40]. In other research, BF-200 ALA-PDT of BCC was highly effective compared to MAL-PDT [73].

For the first time in 1983, PDT was used in the treatment of esophagus cancer. The role of PDT in dysphagia relief and improvement of quality of life is investigated in literatures [33, 61, 64, 68]. In Minamide et al.’s study, talaporfin sodium-PDT divulged better outcomes than porfimer sodium-PDT for local failure after chemoradiotherapy or radiotherapy in esophageal cancer [67].

As in 80–85% of patients with lung cancer, the advanced stage of disease is diagnosed at the referral time, the surgery loses its justification. Neoadjuvant-PDT along with radiotherapy and chemotherapy is an option to improve quality of life in patients [69]. Chemo-photodynamic therapy was suggested by Zhang and his coworkers in treating the primary lung cancer [28, 133]. Shafirstein et al. reviewed the PDT of non-small cell lung cancer (NSCLC). They summarized locally and peripheral tumors, pleural disease and margin control in palliative indications and early stage, and superficial and centrally located endobronchial NSCLC tumors in definitive cases [102]. The pleural membrane of the lung is affected by malignant pleural mesothelioma tumor. In recent years, local control improvement and increasing survival in cooperating PDT and other modalities such as proton therapy suggest encouraging outcomes [69, 96, 103].

In head and neck cancers in addition to surgery, radiotherapy, and chemotherapy, PDT is a good candidate in early-stage diseases, neoadjuvant, intraoperative, and palliative therapy, or in cases of recurrence after treatment [69]. For example, the most important modality in treatment of early-stage oral cavity cancer is surgery, and PDT is an adjuvant treatment for involved margins [66]. Photodynamic therapy with Photofrin was concluded by Hosokawa et al. as useful for treating head and neck carcinoma [35].

Despite insufficient access to photosensitizers with the ability to accumulate in brain tumors as well as light source with appropriate wavelength matched with absorption wavelength of PS, the use of PDT in eliminating the tumor residues and treatment of recurrence cases has been proposed [69]. Intratumoral injection of PSs was suggested by Noske et al. to overcome the blood-brain barrier in PDT of brain tumors, and pre-resection of tumor has an important role to maximize treatment outcome [42, 76].

In single-arm clinical trials, the result of a meta-analysis study confirms that PDT in patients with prostate cancer has short interval between PS administration and illumination, no skin photosensitization, and insignificant impact on erectile and urinary functions [69, 118]. Vascular targeted photodynamic therapy (VTP) was reported as a promising approach in the treatment of low-risk prostate cancer which is associated with increased quality of life [47].

Surgery in early-stage cholangiocarcinoma showed a prolonged survival time in 20–30% patients, and accompanying PDT in the treatment of extrahepatic biliary ducts tumors and nonresectable cholangiocarcinoma leads to more quality of life [65, 85]. Combination of Foscan-PDT with stenting for cholangiocarcinoma reduced the side effects in Kniebühler et al.’s study [48]. The increase in survival created by chemo-photodynamic prompted Gonzalez-Carmona et al. to recommend it in treatment of advanced cholangiocarcinoma [31].

Since the 1980s, the use of PDT in bladder cancer was introduced in carcinoma in situ (CIS) and superficial transitional cell cancer (TCC). Nseyo et al., in a retrospective study, assessed PDT as an effective and safe treatment in 58 patients with resistant TCC and CIS [69, 79]. PDT also represented a less invasive and safe treatment for patients with superficial bladder cancer [13].

High-intensity focused ultrasound (HIFU) and radiofrequency ablation (RFA) are mentioned as two competitors of PDT of breast cancer by Banerjee et al. HIFU possesses the higher ratio of CD4^+^ to CD8^+^ T cell and maybe lower sustained adoptive immune response compare to PDT [9]. In recent years, PDT has remained as an area of interest in palliative treatments and limitation of drug resistance [7]. In this way, outcomes of PDT effectiveness on different tumors are provided in Table 2.

**Table 2:** Summary of PDT of tumors with different photosensitizers and its outcomes

	Photosensitizer	Absorption	Incubation time		References
**Actinic keratosis**	5-Aminolevulinic acid or ALA (Levulan) Methyl aminolevulinate (Metvixia) Aminolevulinic acid hydrochloride (Ameluz)	635 nm 635 nm 635 nm	4–6 h 3 h 3 h	In comparison to cryotherapy, ALA-PDT is superior for individual lesions, and it has better cosmetic effects. MAL-PDT is accompanied with lower pain and better patient tolerance.	[88], [72], [53], [95], [32]
**Squamous cell carcinoma**	5-Aminolevulinic acid or ALA (Levulan) Methyl aminolevulinate (Metvixia)	635 nm 635 nm	4–6 h 3 h	More tumor destruction occurs in MAL (methyl aminolevulinic acid)-PDT compared to cryotherapy and in ALA-PDT compared to 5-FU	[43], [57], [54], [12], [110], [82]
**Basal cell carcinoma**	5-Aminolevulinic acid or ALA (Levulan) Methylene blue	635 nm 640 nm	4–6 h 2 h	Topical imiquimod-PDT is the most effective treatment compared to MAL-PDT. PDT has equal outcome to cryotherapy and surgery	[18], [26], [109], [22], [55], [99], [94], [44]
**Esophageal carcinoma**	Porfimer sodium (Photofrin) 5-Aminolevulinic acid or ALA (Levulan)	630 nm 635 nm	24–48 h 4–6 h	PDT has less morbidity and mortality compared to surgery. PDT is effective treatment of superficial esophageal cancers and approved by FDA as a palliative treatment in symptomatic advanced cancers	[93], [33], [75], [107], [27], [125], [128], [29], [123], [123], [67], [90]
**Lung cancer**	Porfimer sodium (Photofrin) 5-Aminolevulinic acid or ALA (Levulan) N-aspartyl chlorin e6 (NPe6, Laserphyrin)	630 nm 635 nm 664 nm	24–48 h 4–6 h 2–4 h	While surgery remains gold standard of treatment, PDT improves surgical outcomes and can be used as palliative treatment in lung cancer	[93], [112], [17], [62]
**Head and neck, oral cancers**	N-aspartyl chlorin e6 (NPe6, Laserphyrin) Pheophorbide-a Meta-tetra (hydroxyphenyl) chlorin (Foscan)	660 nm 666 nm 652 nm	4–5 h 3 h 48–96 h	Surgery is the best option for tumors > 2–3 mm, and PDT is useful in treatment of early-stage cancers. Oral tissue is preserved in PDT.	[49], [108], [17], [2], [3], [46], [4], [70], [83]
**Glioma**	HPPH: 2-(1-Hexyl-oxyethyl)-2-devinylpyropheophorbide-alpha	665 nm	6 h	PDT and fluorescence-guided surgery is effective in improved survival compared to radiotherapy and surgery	[57]
**Breast**	5-Aminolevulinic acid or ALA (Levulan) Zinc phthalocyanine SnEt2 Purlytin Motexafin lutetium (Lutex) Porfimer sodium (Photofrin) Mono-L-aspartyl chlorin Meta-tetra(hydroxyphenyl) chlorin (Foscan) Verteporfin (Visudyne)	635 nm 675 nm 660 nm 720 nm 630 nm 664–667 nm 652 nm 690 nm	4–6 h 12 h 24 h 3–24 h 24–48 h 4 h 48–96 h 1 h	As a promising modality, more trials need to be conducted in use of PDT for breast cancer	[25], [111], [100], [121], [9]
**Prostate**	Meta-tetra(hydroxyphenyl) chlorin (Foscan) Pd-Bacteriopheophorbide (TOOKAD)	652 nm 763 nm	5 h After a while	Vascular-targeted PDT has approved as a successful focal ablation with minimal side effects	[51], [92], [37], [89], [84], [36]

### Advantages of photodynamic therapy

PDT is cheaper than common radiotherapy and surgery and reduces the post-treatment care time from a few weeks to a few hours. In this modality, irradiation is limited to the treatment region, so there is no photosensitizer activation in obscene of light and there is no cell destruction.

As a noninvasive method, it is a repeatable treatment in the same place. Induction of systemic anti-tumor immunity of PDT is used in the design of antitumor vaccines [30].

Due to the different amount of photosensitizer uptake in normal and neoplastic tissue, fluorescence emission of photosensitizer can be recorded as a noninvasive tumor marker in point monitoring and fluorescence-guided surgery [34].

PDT is generally fitted to superficial lesions; then, it is less efficient in the treatment of large and metastatic tumors. Anyway, with the help of reasonable high-power LEDs and upon excitation by the light source with long wavelength (NIR), the penetration depth increases [104].

### PDT side effects

Pain, erythema, edema, and pustular skin disorder are the most common side effects of PDT along with rare side effects such as urticaria, contact dermatitis, or erosive pustular dermatosis of the scalp (EPDS). In some patients with skin cancer susceptibility, for example in cases with immunosuppression, non-melanoma skin cancer history, or photodamaged skin, the occurrence of basal cell carcinoma (BCC), squamous cell carcinoma (SCC), and melanoma were reported after PDT as consequences of immunosuppression, mutagenesis, and isotopic response [15].

Lehmann summarized the side effects of PDT as follows: pain 92%, erythema/edema 89%, flaking/itching 80%, pustulation 6%, erosion 1.2%, hyper-hypopigmentation 1.0%, and infections (bacterial/viral) 0.3% [86]. To minimize the cutaneous photosensitivity, rapid accumulation in the target tissue and high clearance rate of photosensitizer is desirable (Table 3) [45].

**Table 3:** Photosensitivity of some photosensitizers

Photosensitizer	Skin photosensitivity
5-Aminolevulinic acid or ALA (Levulan)	1–2 days
Methylene blue	1–2 days
Porfimer sodium (Photofrin)	3–8 weeks
Verteporfin (Visudyne)	1–5 days
HPPH: 2-(1-Hexyl-oxyethyl)-2-devinylpyropheophorbide-alpha	6–8 weeks
N-aspartyl chlorin e6 (NPe6, Laserphyrin)	1–2 weeks
Meta-tetra(hydroxyphenyl) chlorin (Foscan)	3–6 weeks

## Conclusions

The clinical use of PDT in therapy dates back to about 40 years ago, but the progress of science in this method has been ahead of the progress of its clinical applications. The effectiveness of PDT in cutaneous malignancies as a promising treatment modality and in some cases such as lung cancer as an adjuvant and palliative method has been proven in lots of literatures.

Although designing methods to overcome constraints such as photosensitivity, poor light penetration, low tumor selectivity, and systemic toxicity seems to be effective in PDT efficiency, more randomized clinical trials are needed to more expanded applications.

Mechanisms of subcellular and tumor localization of photosensitizing agents, as well as of molecular, cellular, and tumor responses associated with photodynamic therapy in conjunction with the technical issues regarding light dosimetry, are really of great importance. Importantly, besides the PDT in cancer treatment, we strongly recommend the role of miRNAs [8] and also stem cell therapy particularly CAR-T cell therapy [6, 98].

In general, it can be said that PDT can have a promising future as a cancer treatment for early diseases or as a synergistic therapy in multimodal oncology.

## Data Availability

The datasets used and/or analysed during the current study are available from the corresponding author on reasonable request.

## Abbreviations

PDT: Photodynamic therapy
PS: Photosensitizer
TPPS: Tetraphenylporphine sulfonate
Pc: Phthalocyanine
AlPcS: Aluminum phthalocyanine sulfonates
mTHPC: Meta hydroxyl-phenyl
MBDs: Membrane-binding domains
PKC: Protein kinase C
PH: Pleckstrin homology
ER: Endoplasmic reticulum
APAF-1: Apoptosis-activating factor-1
DFF: DNA fragmentation factor
PLA2: Phospholipase A2
PLC: Phospholipase C
PGE: Prostaglandin E
CIS: Carcinoma in situ
